# Catechins and Human Health: Breakthroughs from Clinical Trials

**DOI:** 10.3390/molecules30153128

**Published:** 2025-07-25

**Authors:** Elena Ferrari, Valeria Naponelli

**Affiliations:** 1 Laboratory of Biochemistry and Metabolomics, Department of Medicine and Surgery, University of Parma, 43125 Parma, Italy; elena.ferrari@unipr.it; 2 Laboratory of Biochemistry, Molecular Biology and Oncometabolism, Department of Medicine and Surgery, University of Parma, 43125 Parma, Italy

**Keywords:** green tea, polyphenols, flavonoids, catechins, epigallocatechin-3-gallate, clinical trials

## Abstract

Green tea, derived from the unoxidized leaves of *Camellia sinensis* (L.) Kuntze, is one of the least processed types of tea and is rich in antioxidants and polyphenols. Among these, catechins—particularly epigallocatechin gallate (EGCG)—play a key role in regulating cell signaling pathways associated with various chronic conditions, including cardiovascular diseases, neurodegenerative disorders, metabolic diseases, and cancer. This review presents a comprehensive analysis of recent clinical studies focused on the therapeutic benefits and potential risks of interventions involving green tea extracts or EGCG. A systematic literature survey identified 17 relevant studies, classified into five key areas related to catechin interventions: toxicity and detoxification, drug pharmacokinetics, cognitive functions, anti-inflammatory and antioxidant properties, and obesity and metabolism. Findings from these clinical studies suggest that the health benefits of green tea catechins outweigh the potential risks. The review highlights the importance of subject genotyping for enzymes involved in catechin metabolism to aid in interpreting liver injury biomarkers, the necessity of assessing drug–catechin interactions in clinical contexts, and the promising effects of topical EGCG in reducing inflammation. This analysis underscores the need for further research to refine therapeutic applications while ensuring the safe and effective use of green tea catechins.

## 1. Introduction

**Figure 1:**
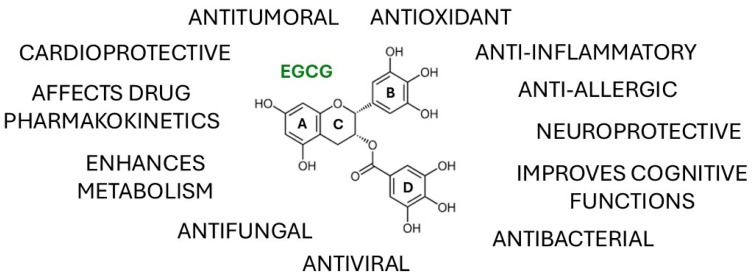
A schematic diagram including the molecular structure of (−)-epigallocatechin gallate (EGCG) and its most widely explored properties.

Tea is among the most ancient and widely consumed beverages worldwide, second only to water. It is produced from the leaves of the *Camellia sinensis* (L.) Kuntze, which belongs to the Theaceae family. Green tea leaves undergo minimal processing when compared with black or oolong tea, a factor that contributes to preserving their antioxidant content [1,2]. Polyphenols are the major antioxidant compounds of green tea, with flavonoids constituting the most abundant class. Catechins (a category of flavanols) are bioactive molecules belonging to the subclass of flavonoids and are the principal secondary metabolites in tea. The catechin content differs depending on the type of tea, with the main components being catechin, epicatechin, epicatechin gallate, epigallocatechin, epigallocatechin-3-gallate, and gallocatechin [1,3]. Epigallocatechin-3-gallate (EGCG), a flavon-3-ol polyphenolic compound, is the most active and abundant natural catechin found in green tea, representing 50–80% of the total catechin content, followed by (−)-epigallocatechin (EGC), (−)-epicatechin gallate (ECG), (−)-epicatechin (EC), and (+)-catechin (C) [4,5,6]. The chemical structure of EGCG (Figure 1) consists of two benzene rings (A and B), a dihydropyran heterocyclic ring (C), and a gallate moiety (D) [7].

The therapeutic potential and adverse effects of catechins or EGCG have been investigated in decades of preclinical studies, including both in vitro and in vivo experiments, as well as clinical trials [8]. Although catechins, particularly EGCG, have emerged as potential therapeutic agents, clinical trials are still essential to validate their efficacy and safety and to optimize their delivery to target tissues. Indeed, the clinical approach is of instrumental significance in improving health outcomes and potentially extending lives.

Here, we provide a comprehensive review of the cutting-edge clinical studies published in recent years and based on green tea extracts or EGCG interventions. To provide a coherent foundation for the analysis, we first outline (i) the molecular targets, bioavailability, and safety profile of EGCG, and (ii) the thematic domains that informed the categorization of the included studies.

### 1.1. Epigallocatechin Gallate: Molecular Targets, Bioavailability, and Safety Considerations

#### 1.1.1. Molecular Targets

Green tea catechins, especially EGCG, modulate a plethora of cellular signaling pathways involved in the onset and progression of several chronic pathologies, including cardiovascular, neurodegenerative, and metabolic diseases, and cancer (Figure 1) [9,10,11,12,13,14,15].

EGCG has demonstrated considerable therapeutic potential through a multifaceted mechanism of action, chiefly by modulating key signaling pathways such as JAK/STAT, NF-κB, AKT, and Notch. This regulatory capacity underscores its pivotal role in governing essential cellular processes, including apoptosis, proliferation, and survival. By mitigating oxidative stress, dampening inflammatory responses, and promoting programmed cell death, EGCG has emerged as a promising candidate for the treatment of various pathological conditions [16,17].

EGCG targets the JAK/STAT and Notch pathways to reduce oxidative stress. EGCG has been shown to inhibit STAT3 phosphorylation, thereby disrupting JAK/STAT signaling, which is critical for cell proliferation, survival, and inflammation. EGCG modulates Notch signaling, particularly Notch-1, which is involved in inflammatory and oxidative stress responses. It has been shown to attenuate oxidative stress by modulating several key biomarkers. These include: malondialdehyde (lower levels indicate reduced lipid peroxidation); glutathione (GSH/GSSG higher levels reflect enhanced redox balance); glutathione peroxidase (higher activity supports ROS scavenging); superoxide dismutase (higher activity contributes to ROS neutralization); catalase (higher activity improves H_2_O_2_ breakdown) [17].

EGCG has been shown to modulate tumor-driven signaling cascades, promoting programmed cell death selectively in abnormal cells while sparing healthy tissue. This selective cytotoxicity is mediated through key molecular targets, including NF-κB, PI3K/Akt, p53 and 67LR [18]. Moreover, EGCG suppresses TGF-β1-induced epithelial–mesenchymal transition, which is a critical process in cancer metastasis and fibrotic progression [19]. By interfering with this transition, EGCG demonstrates promising therapeutic potential in oncology and beyond.

According to Chourasia et al., computational models have revealed that EGCG can effectively bind to SARS-CoV-2’s papain-like protease (PLPro). This interaction has the potential to disrupt viral replication and hinder immune evasion strategies [20]. The authors also highlight EGCG’s ability to modulate pro-inflammatory cytokines, such as IL-1β and IL-6. Considering EGCG’s impact on oxidative stress, inflammation, and viral entry, it emerges as a potential candidate for preventive and therapeutic applications against SARS-CoV-2. In contrast, Bimonte et al. adopt a more cautious position. They argue that EGCG should not be considered a definitive therapeutic option for COVID-19 due to its lack of molecular specificity, which raises concerns about off-target interactions. The potential for EGCG to bind off-target proteins could lead to unintended side effects. The authors therefore suggest that EGCG may be more appropriate as a nutraceutical or dietary supplement, particularly in the early stages of clinical manifestation, rather than as a primary pharmacological intervention [21].

#### 1.1.2. Bioavailability

EGCG holds therapeutic promise but is hindered by poor systemic availability and physicochemical instability. Its fate in the body depends on absorption, distribution, metabolism, and excretion. EGCG is absorbed in the small intestine, but its hydrophilic nature and low membrane permeability restrict uptake [22]. The compound is chemically unstable at neutral or alkaline pH and elevated temperatures, leading to degradation prior to absorption. Additionally, food intake impairs absorption, while fasting conditions enhance plasma concentrations [23]. The gut microbiota further transforms EGCG into ring-fission metabolites, which may exert distinct biological effects [24]. Following absorption, EGCG is distributed to various organs. However, liver cells rapidly metabolize the remaining EGCG into methylated, sulfated, and glucuronidated conjugates, which are less bioactive. EGCG is rapidly excreted through urine and bile, with a short biological half-life and minimal tissue accumulation [8]. As a result, less than 1% of orally ingested EGCG reaches systemic circulation in its active form. Finally, EGCG bioavailability exhibits high interindividual variability, influenced by gastrointestinal absorption efficiency, molecular stability under digestive conditions, nutritional context, and type of administration [25]. Topical formulations of EGCG bypass gastrointestinal degradation and first-pass metabolism, enabling direct action at the target site. This localized delivery helps explain its consistent efficacy in dermatological contexts, in contrast to the variable systemic outcomes observed with oral dosing [26].

To address EGCG’s limited bioavailability, innovative delivery systems—particularly lipid-based and polymeric nanoparticles—are showing promise [6,27]. Lipid carriers such as liposomes, solid lipid nanoparticles (SLNs), and nanostructured lipid carriers (NLCs) protect EGCG from degradation, improve absorption, and enable targeted tissue delivery. Liposomes offer a versatile platform for co-delivering EGCG alongside chemotherapeutic agents, enhancing EGCG’s bioavailability and synergistically increasing therapeutic efficacy [28,29]. Polymeric nanoparticles, particularly those composed of poly(lactic-co-glycolic acid) (PLGA), chitosan, and PEG-based frameworks, provide controlled release, enhanced solubility, and improved physicochemical stability of EGCG [30].

#### 1.1.3. Evidence and Variables of Hepatotoxicity

Research findings on hepatotoxicity associated with green tea catechins are inconsistent and inconclusive, highlighting the pharmacokinetic complexity and inter-individual variability in response. A comprehensive review of the main studies that have investigated the toxic effects of green tea extracts on liver function is provided elsewhere [31]. The extent of liver injury appears to depend on multiple factors, including the administered dose, formulation type, study duration, and characteristics of the population examined. Most observed adverse liver events were mild to moderate in severity, while severe hepatic necrosis associated with oxidative stress was reported in only a small proportion of individuals [31,32]. Thirteen serious adverse events were reported in five studies resulting from the consumption of at least 800 mg/day of EGCG from green tea extract (GTE) or Polyphenon E (Table 1) [33,34,35,36,37].

Across all dosage forms, none of the studies reviewed by Hu et al. reported adverse hepatic effects at EGCG doses equivalent to or below 676 mg/day [31]. Among studies utilizing a beverage or food-based dosage form, one of the highest reported intakes involved a green tea extract (GTE) delivering 1519.7 mg/day of total catechins and 704 mg/day of EGCG, administered in three divided doses before meals. No hepatic adverse events were observed, irrespective of whether subjects were in a fed or fasted state [38]. In contrast, when EGCG was administered at 843 mg/day, no cases of severe hepatotoxicity were reported among subjects consuming a green tea-based beverage, whereas a higher incidence of liver enzyme elevations was observed in those receiving the same dose as a bolus in capsule form [36]. Changes in liver biomarkers were observed after only 10 days in healthy male subjects consuming 800 mg/day of EGCG on an empty stomach [39]. In contrast, when EGCG was taken during or after meals, or administered in divided doses throughout the day, hepatotoxicity typically emerged in studies lasting 60 days or longer [33,34,35,36,37,40,41]. All adverse events returned to normal after treatment was discontinued.

The variability in the effects observed across studies may be attributable, at least in part, to the inclusion of heterogeneous populations, such as individuals with non-alcoholic fatty liver disease, diabetes, multiple sclerosis, cancer, uterine fibroids, metabolic syndrome, hypertension, and hypercholesterolemia [42].

**Table 1:** Hepatotoxicity and dosage data from green tea extract trials.

Population	Catechin Dose	Treatment Period	Hepatotoxicity (Cases/Grade) *	Reference
Breast cancer patients (n = 30 women)	2–4 capsules Polyphenon E (1600 mg EGCG/day)	6 months	2/grade 1 1/grade 3	Crew et al. (2012) [33]
Chronic lymphocytic leukemia patients (n = 42)	5 or 10 capsules Polyphenon E (2000 mg EGCG and 4000 mg EGCG/day) with meals	6 months	13/grade 1 6/grade 2 1/grade 3	Shanafelt et al. (2013) [34]
Healthy women (n = 41)	Capsules of Polyphenon E (800 mg EGCG/day) with meals	4 months	1/grade 3 (9 with elevated enzymes)	Garcia et al. (2014) [35]
Postmenopausal women (n = 799)	843 + 44 mg/day GTE capsules (n = 400) vs. placebo (n = 399) with meals	12 months	43/grade 1 (4 placebo group) 7/grade 2 6/grade 3 1/grade 4	Dostal et al. (2015) [36]
Multiple sclerosis patients (n = 13)	2 capsules Polyphenon E (800 mg/day EGCG) (n = 8) vs. placebo (n = 5) with meals	6 months	1/grade 3	Lovera et al. (2015) [37]
Multiple sclerosis patients (n = 7)	2 capsules Polyphenon E (800 mg/day EGCG) with meals	1 year	4/grade 1 1/grade 4	Lovera et al. (2015) [37]

* For the classification of liver toxicity, National Cancer Institute Common Terminology Criteria for Adverse Events (NCI CTCAE) are often applied. The Drug-Induced Liver Injury Network (DILIN) developed a 5 point scale (1, mild; 2, moderate; 3, moderate to severe; 4, severe; 5, fatal) for grading the severity of liver injury based upon the presence of jaundice, hospitalization, signs of hepatic or other organ failure, and ultimate outcome [43].

#### 1.1.4. Long-Term Safety

While EGCG is generally considered safe at recommended doses for short-term use, long-term safety data beyond this duration remains limited. Most human clinical trials span 8–12 weeks, with only a few extending up to 6–12 months. Clinical evidence indicates that individual tolerance varies, influenced by factors such as genetic polymorphisms and underlying health conditions, as previously reported [44]. As a result, recent studies recommend establishing personalized dosing guidelines based on patient-specific characteristics to minimize adverse outcomes. Moreover, current research highlights the importance of identifying biomarkers for early detection of EGCG-induced toxicity, which could pave the way for safer long-term use in therapeutic contexts [45].

## 2. Main Topics Informing Clinical Study Categorization

As previously noted, green tea catechins—particularly the predominant compound epigallocatechin gallate (EGCG)—have been linked to adverse effects, including gastrointestinal irritation and hepatotoxicity [31,46,47]. Notably, these effects have been frequently observed with highly concentrated tea supplements [43]. However, it is challenging to predict the extent to which high doses of catechins or EGCG will cause hepatotoxicity in an individual, given the genetic variability that affects how people respond to these compounds. For example, genetic variants in the catechol-O-methyltransferase (COMT) gene may affect the metabolic processing of these compounds, thus modulating the individual’s response to catechins [48].

Catechins have been shown to affect the efficacy of drugs by altering their metabolism. While green tea catechins exert only a limited effect on cytochrome P450-mediated drug metabolism [49], they have demonstrated a more pronounced impact on drug transporters and drug-metabolizing enzymes [50]. Their ability to inhibit the organic anion-transporting polypeptide (OATP) transporters and P-glycoprotein (ABCB1 transporter) has been demonstrated in both in vitro and in vivo experiments [51,52,53]. Moreover, green tea catechins have also been shown to interfere directly with certain drugs. Research has shown that EGCG can antagonize the effects of Bortezomib, a proteasome inhibitor used in cancer therapy, thereby blocking its action [54].

Green tea catechins, especially EGCG, are powerful antioxidants. They help neutralize free radicals—unstable molecules that can cause oxidative stress and lead to molecular and cellular damage, aging, and various diseases [8]. The hydroxyl groups of EGCG are responsible for its strong antioxidant activity (via electron-donating capacity). This enables the molecule to scavenge free radicals or chelate metal ions, thereby preventing heavy metal toxicity [55,56,57].

EGCG exerts antitumoral properties via multiple mechanisms, including antioxidant and antiangiogenic effects, blocking of cell cycle progression, induction of autophagy or apoptotic cell death, and alteration of DNA and microRNA expression [8,22]. Furthermore, EGCG is recognized for its anti-inflammatory properties, which are achieved by modulating key signaling pathways. For example, it has been demonstrated that EGCG exerts anti-inflammatory effects by suppressing NF-kB activation and reducing the release of inflammatory mediators [9].

Inflammation is a key process involved in the pathogenesis of numerous chronic diseases, including neurodegenerative conditions. Evidence from in vivo and in vitro studies suggests that catechins, and particularly EGCG, may have a role in alleviating neurodegenerative diseases such as Alzheimer’s, Parkinson’s, Huntington’s, and multiple sclerosis, as well as cognitive deficits [58]. Two studies have assessed the association between the level of dietary intake of catechins and cognitive function/health in healthy individuals. Kesse-Guyot et al. found an association between 13 years of dietary catechin intake and improvements in language and verbal memory scores and reductions in executive functioning factor in 2574 adults; Biasibetti et al. observed an inverse association between dietary intake of catechins and impaired cognitive status in 808 Italian individuals aged over 50 years [59,60]. More recently, a randomized controlled study reported that the daily intake of green tea catechins may improve working memory in middle-aged and older subjects [61]. Interestingly, other natural compounds such as coumarins and isocoumarin derivatives have exhibited neurotrophic, antioxidant, and anti-inflammatory properties in preclinical studies [62,63]. Despite their distinct structures and biosynthetic origins, both EGCG and isocumarin derivatives manifest overlapping bioactivities that could justify a comparative or combinatorial evaluation.

Finally, green tea catechins may play a preventive role against obesity. Several clinical studies have demonstrated the beneficial effects of green tea on weight loss in overweight and obese individuals. However, tea has sometimes had no significant effect on weight loss. This may be due to the poor bioavailability of tea bioactive compounds or the heterogeneity in individual responses to tea consumption [64].

In recent years, various studies have shown that tea polyphenols can significantly reduce serum levels of triacylglycerols, total cholesterol, and LDL-C, while increasing levels of HDL-C in patients with hyperlipidemia, suggesting a protective effect on vascular endothelial function [65,66].

**Figure 2:**
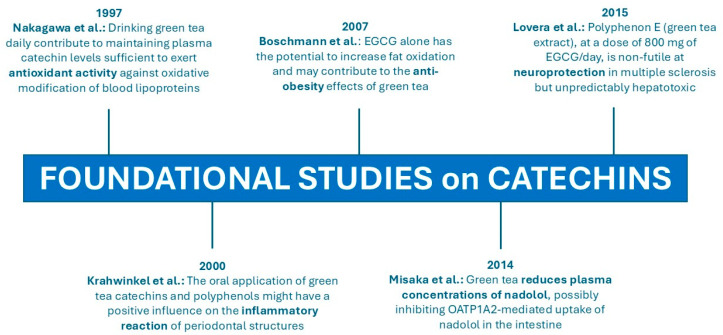
The clinical trials that laid the groundwork for current clinical research [37,52,67,68,69].

Figure 2 presents the selected human studies that constitute the foundational framework for the issues discussed above [37,52,67,68,69].

There are still unresolved issues impacting the clinical use of tea polyphenols. The primary concerns are dosage, specificity, potency, feasibility, and the potential for adverse effects. In the contemporary context, large-scale cohort and intervention studies remain essential.

## 3. Major Findings from the Clinical Trials Reviewed

A comprehensive search of the PubMed database was conducted to identify relevant clinical studies investigating the effects of epigallocatechin gallate. We selected PubMed as our primary source due to its extensive coverage of biomedical literature and the inclusion of MEDLINE-indexed records. These features enhance the retrieval of clinically relevant studies and support the comprehensive exploration of health-related research, including clinical trials. The search was performed using the keyword “epigallocatechin gallate” and refined by applying the following filters: article type—clinical trials and randomized controlled trials; publication date range—2022 to 2024; and availability—full-text accessible articles. This approach yielded 17 peer-reviewed interventional studies, all of which were included in the present review. No exclusion criteria were applied to the retrieved records at this stage. The selected timeframe aims to capture recent EGCG-based interventions within the context of current clinical practice and patient populations, synthesizing emerging evidence through a narrative review framework.

These were categorized according to the effects produced and the compounds tested, as follows: toxicity and detoxification effects of catechins; effect of catechins on drug pharmacokinetics; effect of catechins on cognitive functions; anti-inflammatory and antioxidant effects of EGCG; effect of EGCG on obesity and metabolism. The following subsections outline the purposes, types of intervention, and key findings of the reviewed studies.

### 3.1. Toxicity and Detoxification Effect of Catechins

The hepatic safety profile of green tea extract (GTE) was investigated by Siblini et al. (Table 2) [70]. Before launching a study to evaluate the effectiveness of EGCG in improving fertility in women with unexplained infertility and uterine fibroids, the possible effects of a green tea extract given in combination with clomiphene or letrozole (ovarian stimulation medications) on liver function tests were assessed. A daily dose of GTE containing 720 mg of EGCG was taken in a fed state for at least one month, with or without medication, for 5 days. No significant changes in liver function tests (ALT, AST, direct bilirubin, total bilirubin, mid-cycle endo thickness, folate) were observed throughout the study, and no subjects were found to have folate deficiency by the end of the study.

In the article by Acosta et al. (Table 2), the hepatic response to long-term, high-dose GTE was investigated in postmenopausal women through the lens of nutritional genetics [71]. Genotyping for the catechin-metabolizing enzymes catechol-O-methyltransferase (COMT) and uridine 5′-diphospho-glucuronosyltransferase 1A4 (UGT1A4) proved to be informative for individuals on high-dose GTE supplementation. In fact, subjects with the UGT1A4 (rs6755571) A/C genotype could experience significant increases in ALT concentrations from baseline to months 6 and 9 compared to the UGT1A4 (rs6755571) C/C genotype; subjects with the COMT (rs4680) A/A genotype could experience significant decreases in the AST/ALT ratio from baseline to month 3 compared to the COMT (rs4680) A/G genotype. A secondary analysis conducted in a subset of the same postmenopausal women showed that the supplementation was not associated with a reduction in plasma F2-isoprostanes, biomarkers of oxidative stress (Table 2) [72]. In addition, the COMT genotype did not alter the effect of GTE on the F2-isoprostanes levels in the interventional group.

The study by Wan et al. (Table 2) [73] investigated the protective effect of tea polyphenols (TP) against the toxicity of acrylamide exposure in vivo. Their results suggest that TP may promote urinary excretion of acrylamide via the oxidative pathway that occurs when acrylamide is converted to glycidamide. Following the ingestion of acrylamide (via a specific dose of potato chips) and TP intervention, the excretion of mercapturic adduct of glycidamide was increased. TP promoted the conjugation of the sulfur of glutathione with the electrophilic groups of glycidamide, thus accelerating the excretion of glycidamide as N-(R,S)-acetyl-S-(2-carbamoyl-2-hydroxyethyl)l-cysteine, the mercapturic adduct of glycidamide [74,75]. This, in turn, led to lower levels of hemoglobin adducts of glycidamide in vivo.

### 3.2. Effect of Catechins on Drug Pharmacokinetics

According to Misaka et al. (Table 3), GTE dissolved in water (containing 300 mg of EGCG) significantly reduced plasma concentrations and urinary excretion of (unmodified) fexofenadine compared with water in healthy volunteers [76]. Co-administration of GTE was shown to reduce exposure to fexofenadine, putatively by inhibiting its OATP1A2-mediated intestinal absorption, demonstrating that green tea should not be consumed during anti-allergic therapy with fexofenadine. This effect was not observed in the case of pseudoephedrine.

A randomized, two-period, cross-over study investigated whether concomitant administration of green tea extract and nintedanib (a small-molecule kinase inhibitor) in patients with fibrotic interstitial lung disease could result in a clinically relevant herb–drug interaction (Table 3) [77]. The study reported a significant decrease in nintedanib exposure when co-administered with green tea extract. The interaction between nintedanib and GTE is hypothesized to be based on the induction of the efflux transporter ABCB1, which is responsible for drug excretion. This effect was predominant in patients with the wild-type ABCB1 transporter compared to those with the heterozygous single-nucleotide variant.

### 3.3. Effect of Catechins on Cognitive Functions

According to Uchida et al. (Table 4), consumption of matcha green tea powder, which contains bioactive compounds such as catechins, theanine, and caffeine, has beneficial effects on emotional perception and sleep quality in older adults with mild cognitive decline [78]. This observation suggests the potential for a daily routine to enhance cognitive function and prevent dementia.

The study by Cieuta-Walti et al. (Table 4) is a multicenter, randomized, and controlled phase Ib study, in which children with Down syndrome have been enrolled [79]. The main outcomes were safety and cognitive and functional performances. Catechin treatment was well tolerated by patients, with a similar rate of adverse events observed in control and treatment groups. No instances of cardiac, renal, or hepatic toxicity, nor neurophysiological changes, were observed. However, the EGCG group did not demonstrate improvements in cognitive and functional outcomes compared to the placebo group.

### 3.4. Anti-Inflammatory and Antioxidant Effects of EGCG

In the study by Zeng et al. (Table 5), a topical EGCG solution was found to be effective in reducing periodontal inflammation in patients with periodontitis [80]. A 5 mg/mL EGCG solution was used to replace the distilled water in the ultrasonic scaler during periodontal therapy on the test side of each patient’s mouth. The control side underwent standard periodontal care. The clinical parameters of periodontitis generally improved after treatment on both sides of the mouth. The probing depth, clinical attachment level, gingival index, and plaque index showed no statistical difference (*p* > 0.05) between the two side groups at 6 and 12 weeks post-treatment. However, at 12 weeks, the mean reduction in bleeding index was significantly greater on the test side than on the control side.

According to Zhao et al. (Table 5), prophylactic use of EGCG solution (660 μmol/L) significantly reduced the incidence and severity of radiation-induced dermatitis (RID) in patients receiving adjuvant radiotherapy for breast cancer [81]. EGCG solution turned out to be a promising treatment for RID also in patients diagnosed with thoracic cancer undergoing radiotherapy (Table 5) [82]. It was dissolved in increasing concentrations ranging from 660 to 2574 µmol/L and sprayed into the irradiated field. The application of the EGCG solution led to a significant reduction and improvement in grade III RID symptoms, with no adverse events reported.

The study by Yin et al. (Table 5) is a prospective phase I/II clinical trial in which patients with cancer and COVID-19-induced pneumonia were treated with nebulized inhalation of an EGCG solution [83]. The safety analysis was performed during the phase I trial, in which the EGCG dose escalation stopped at 8817 µmol/L. The highest dose at which no adverse events were observed (5878 µmol/L) was the recommended dose for the phase II trial. After one week of treatment, a computed tomography scan showed that the incidence of non-progression of pneumonia was 82%, and the rate of improvement of pneumonia was 56.4%. However, laboratory parameters related to inflammation (white blood cell count, lymphocyte count, IL-6, ferritin, C-reactive protein, and lactate dehydrogenase) were not significantly different before and after treatment. The authors speculate that the inflammatory microenvironment of the tumor patients may have accounted for the non-significant changes in inflammatory indices.

In idiopathic pulmonary fibrosis, EGCG exposure appeared to attenuate the activity of multiple pro-inflammatory and stress effectors [84]. Cohen et al. (Table 5) conducted a detailed investigation of the molecular and cellular basis of interstitial lung disease (ILD) using single-cell RNA-Seq on tissue biopsies. The results indicate that fibroblasts from ILD patients with mild disease undergoing diagnostic biopsy have higher levels of TGF-β1 and pro-inflammatory pathway signaling than either non-diseased donor tissue or end-stage ILD explant tissue. However, gene expression analysis revealed that EGCG administration inhibited TGF-β signaling in ILD fibroblasts, and this inhibition attenuated profibrotic signaling at the protein level.

### 3.5. Effect of EGCG on Obesity and Metabolism

The study by Wilasrusmee et al. (Table 6) was based on obese subjects and demonstrated that, following eight weeks of EGCG treatment, systolic blood pressure (SBP), diastolic blood pressure (DBP), and mean arterial pressure (MAP) significantly decreased, while the power ratio of low-frequency to high-frequency (LF/HF ratio) significantly increased, indicating a shift toward sympathetic dominance [85]. Notably, the study also observed a negative correlation between high-density lipoprotein cholesterol and SBP, DBP, and MAP, underscoring its role in protecting against hypertension.

In the study by Gu et al. (Table 6), the effect of EGCG on obesity-related precocious puberty was investigated at the molecular level using an integrated metabolomics and network pharmacology approach [86]. Serum metabolomics, performed using high-performance liquid chromatography-electrospray ionization ion-trap tandem mass spectrometry, revealed that EGCG alters the serum metabolome. According to the differential metabolites in obese girls, EGCG may have a regulatory effect on serum lipid metabolism, with possible consequences for hormonal disorders. The integration of metabolomics and network pharmacology has identified six hub targets, namely AKT1, EGFR, ESR1, STAT3, IGF1, and MAPK1, that may be critical for the effects of EGCG on obesity-related precocious puberty.

Jardon et al. (Table 6) tested whether polyphenols could modify the gut microbial composition (by acting as prebiotics) and/or selectively inhibit potentially pathogenic species often associated with metabolic disorders in overweight or obese individuals [87]. Supplementation with EGCG plus resveratrol did not alter the gut microbiota composition of men and women. However, microbiota composition could predict polyphenol-induced changes in mitochondrial respiration (measured ex vivo in permeabilized skeletal muscle fibers from biopsies) in men, but not in women. This was linked to a reduced abundance of potent microbial producers of short-chain fatty acids in men. These findings suggest a sex-specific relationship between microbiota and metabolic health.

The study by Churm et al. (Table 6) investigated the effect of acute ingestion of EGCG on catecholamines, catecholamine metanephrines, and systemic metabolic and cardio-respiratory variables during continuous incremental cycling exercise to exhaustion [88]. Oxidative energy expenditure was determined by means of indirect calorimetry. EGCG ingestion reduced circulating catecholamine concentrations and peak lipid oxidation in response to graded exercise.

## 4. Discussion

The reviewed studies aim to evaluate the impact of green tea extracts, tea polyphenols, matcha, or simply EGCG on health-related outcomes. In terms of statistical design, eleven trials were randomized and placebo-controlled [71,72,73,78,79,80,81,85,86,87], while three trials used a two-period crossover design, where each participant received treatment and placebo in a randomized order over two periods [76,77,88]. The latter approach minimized the impact of individual variability, with participants serving as their own control. Choen et al.’s study was reported as pilot study [84]. Three trials were reported as phase I [70,79,82], designed to assess the safety and efficacy of the treatment in a relatively small group of patients; one trial was reported as phase I/II [83], with a safety analysis in phase I and a safety and efficacy analysis in phase II; and three trials were reported as phase II [71,81,85], designed to evaluate the reduction in specific symptoms in a large number of patients.

All reviewed studies reported on the treatment formulation, administration, frequency, and duration. The studies that employed green tea extract, tea polyphenols, matcha or a food supplement as a treatment [70,71,72,73,76,77,78,79] specified the percentage of EGCG content; only three of them also quantified the total catechin content [72,73,78]. The studies that used EGCG in solution or capsule [80,81,82,83,84,85,86,87,88] specified its dosage; some of them also reported the supplier and the purity of the EGCG extract [80,81,82,83,85,88].

Although green tea has shown promise as an adjunctive therapy for numerous chronic health conditions, fears of hepatotoxicity have prevented its widespread clinical use. The first studies on EGCG toxicity, particularly hepatotoxicity, date back to the early 2000s [89,90,91]. More recently, the EFSA’s Panel on Food Additives and Nutrient Sources considered the possible association between EGCG (from dietary sources) and hepatotoxicity [43]. It was observed that the average daily intake of EGCG from green tea infusions ranges from 90 to 300 mg/day, whereas the exposure of high consumers is estimated to reach 866 mg EGCG/day in the adult EU population. The Panel concluded that catechins from green tea infusions or reconstituted beverages are generally regarded as safe, provided that intakes are consistent with reported intakes in European Member States. However, rare cases of liver injury have been reported following the consumption of green tea infusions. These events are likely attributable to idiosyncratic responses, influenced by factors such as genetic predisposition, nutritional or fasting status, and overall liver health [43,92].

In line with the EFSA’s observations, Siblini et al. found that drug-induced liver injury was not associated with a daily dose of 720 mg EGCG taken for at least one month in healthy women of reproductive age [70]. Instead, Acosta et al. identified a risk factor for liver injury from high-dose GTE [71]. Their study provides insight into the discussion of genetically guided approaches to the therapeutic use of GTE, demonstrating that the UGT1A4 (rs6755571) A/C genotype may be a risk factor for clinically relevant serum transaminase elevations after 6–9 months of high-dose GTE supplementation in postmenopausal women.

Acrylamide is a global health concern because it is commonly used in water purification, cosmetics, and plastics. Additionally, it is a byproduct of the Maillard reaction in carbohydrate-rich, heat-processed foods [93,94]. Concerning the detoxification effect of catechins, the results by Wan et al. indicate that tea polyphenols promote the oxidative metabolism of acrylamide [73]. In fact, TP accelerated the elimination of the acrylamide-derivative glycidamide via the glutathione S-transferase pathway and the oxidative pathway for further glycidamide conversion [74]. However, the mechanism of action of catechins on the altered metabolic pathway of acrylamide is still unclear and deserves further investigation.

Green tea and its catechins are acknowledged to interact with hydrophilic drugs [95]. Studies on the effect of green tea consumption on the pharmacokinetics of drugs have mainly been carried out with statins, cardiovascular drugs, and anticancer drugs [96,97]. By reducing systemic drug exposure, these interactions may reduce therapeutic efficacy, particularly for drugs with defined and narrow therapeutic ranges. The articles by Misaka et al. and Veerman et al. confirmed a reduction in exposure to fexofenadine and nintedanib when administered with GTE, providing valuable recommendations for patients and clinicians [76,77]. Whether ethnic differences influence the pharmacokinetic interactions between drugs and catechins remains uncertain, yet it continues to be a relevant topic.

Topical EGCG delivery systems are expected to avoid the risk of toxicity due to EGCG accumulation while maintaining the anti-inflammatory and antioxidant effects. In the clinical study by Zeng et al., an EGCG solution was employed as a local coolant during scaling and root planing procedures in periodontitis patients, resulting in a reduction in the gingival bleeding index [80]. As recently reviewed, the effects of EGCG on periodontal diseases include the inhibition of various periodontal pathogens and the expression of anti-inflammatory and antioxidant effects, thus mitigating the damage to periodontal tissues and promoting the regeneration of periodontal tissues [7]. It has been demonstrated that EGCG exerts a positive effect also in the treatment of various dermatological conditions, including psoriasis, atopic eczema, and UV-induced skin damage [98]. Furthermore, the topical administration of EGCG solutions has demonstrated efficacy in dermatological patients with compromised health due to cancer and radiation therapy. As reported in two included studies, the prophylactic effect of an EGCG solution provided a well-tolerated and valid option for cancer patients at risk of RID due to radiotherapy [81,82]. Inhalation of EGCG solution via an aerosol delivery system was also well tolerated by cancer patients, demonstrating that EGCG may be effective in COVID-19-induced pneumonia [83]. Notably, the authors of the mentioned studies specified that the EGCG solution was freshly prepared [80,81,82]. A substantial body of literature exists on the stability of EGCG solution, and it is well-documented that an increase in temperature and pH, as well as a decrease in concentration, have a detrimental effect on the stability of EGCG [98]. Purity testing of the EGCG solution should be conducted prior to its application as a treatment to ensure its quality and efficacy.

Green tea and matcha have often been promoted for their potential to aid weight loss and body fat reduction in the context of obesity and weight management [99]. Nevertheless, a consensus on green tea has proven elusive, given the heterogeneity of the scientific results. Key factors contributing to this variability include study design, dosage, extract type, treatment duration, and sample selection. A recent review and meta-analysis showed that green tea supplementation is likely to be associated with a reduction in body weight and BMI in obese patients, for periods longer than 12 weeks and at doses <800 mg/day [100]. However, it is important to note that its major catechin, EGCG, does not function as a panacea for weight loss. Rather, green tea supplementation can be employed as a complementary measure to a comprehensive approach to weight management, including a healthy diet and regular exercise [8,100]. As demonstrated in the study by Wilasrusmee et al., the cardiovascular health of obese subjects improved after eight weeks of EGCG treatment [85]. The potential mechanisms behind these observations may include the direct effect of EGCG as a sympathetic potentiator or a compensatory response following its vasodilatory effect.

The relationship between the gut microbiota and EGCG is an emerging area of research, with evidence of a mutual interaction [101,102]. In light of the conclusions of Jardon et al., future studies investigating the interaction between gut microbiota and host metabolism should take into account gender and metabolic and microbial phenotypes. This approach will facilitate the development of personalized interventions based on polyphenol supplementation to improve metabolic health in overweight individuals [87].

The consumption of green tea has been demonstrated to have a potential role in the reduction in cognitive impairment, as evidenced by numerous epidemiological studies. Catechins have been shown to possess antioxidant, anti-inflammatory, and neuroprotective properties against neurological disorders [4,103]. A previous pilot study demonstrated that EGCG, an inhibitor of dual-specificity tyrosine-(Y)-phosphorylation regulated kinase 1A (DYRK1A), administered to young patients with Down syndrome improved visual recognition memory, working memory performance, and adaptive behavior [104]. Among the articles reviewed, Uchida et al. found an improvement in social cognition and sleep quality associated with matcha consumption, which may lead to improvements in cognitive function [78]. Instead, as reported by Cieuta-Walti et al., an EGCG-treated group with Down syndrome showed no improvement in cognitive and functional outcomes [79]. The authors of this study propose several potential explanations for the absence of treatment efficacy, including the following: the dose of EGCG administered is insufficient to achieve pharmacologically active concentrations, and EGCG concentrations fall below pharmacologically active levels during the night, when new information is consolidated. As a result of oral intake, the concentration of EGCG in human plasma is very low, with EGCG disappearing from the systemic circulation within a few hours due to rapid and extensive metabolism (methylation, glucuronidation, and sulfation) [4]. In other words, the limited bioavailability and inconsistent pharmacokinetic profile of EGCG may result in substantial variability in catechin absorption and metabolism between individuals, which may account for the observed results. Furthermore, the neuropsychological tests may not have been adequate for detecting an EGCG-mediated effect. Studies have shown that gut microbial metabolism generates a variety of EGCG ring fission metabolites, which have been detected in both plasma and urine [105]. These compounds exhibit increased bioavailability and the ability to cross a blood–brain barrier model, as shown for intact EGCG [106,107]. Emerging evidence suggests that microbial ring fission metabolites may play a role in mitigating brain dysfunction. However, interindividual variability in gut microbiota composition is expected to significantly affect the absorption and systemic availability of these compounds [4].

## 5. Conclusions

The clinical studies reviewed have confirmed that green tea catechins continue to offer more promise than pitfalls. Their findings underscore the significance of (i) subject genotyping for catechin-metabolizing enzymes in the interpretation of liver injury biomarkers [71]; (ii) assessing drug exposure to identify potential interactions with co-administered green tea extract [76,77]; and (iii) the topical application of EGCG solutions to mitigate inflammation [80,81,82,83].

Advancements in genotyping and personalized medicine are providing deeper insight into the interindividual variability observed in response to EGCG treatment. Genetic differences, particularly in genes involved in catechin metabolism and transport, may significantly influence the pharmacokinetics and pharmacodynamics of EGCG. Integrating genotypic data into therapeutic design could facilitate the development of personalized EGCG-based interventions with enhanced efficacy. This approach lays the foundation for precision nutrition strategies tailored to individual genetic profiles.

Growing evidence highlights the gut microbiota as a key modulator of EGCG’s biotransformation and biological activity. The interaction between catechins and gut microbiota is pivotal in elucidating the mechanisms through which these compounds can exert substantial health effects despite their limited bioavailability [108]. Variations in microbiota composition can influence catechin metabolism and may partially explain the heterogeneity observed in individual treatment responses. Recognizing host–microbiota interplay is therefore essential for optimizing EGCG-based therapeutic strategies. This research area opens avenues for the development of targeted dietary supplements aimed at enhancing the physiological benefits of catechins through microbiota-directed approaches.

Future clinical trials and applications could benefit from strategies that enhance EGCG bioavailability. These include the use of high-purity EGCG extracts, the administration under fasting conditions, the use of nanostructure-based delivery systems, and the co-administration with bioactive agents such as ascorbic acid, omega-3 fatty acids, or piperine [109,110]. Finally, monitoring EGCG plasma concentrations during interventional trials can facilitate correlation analyses between EGCG exposure and measured outcomes.

## Figures and Tables

**Table 2:** Toxicity and detoxification effect of catechins: evidence from interventional clinical studies.

Author (Year)	Country	Purpose	Trial Registry Number	Sample	Intervention	Major Findings
Siblini et al. (2023) [70]	U.S.A.	To assess the hepatic safety profile of EGCG in reproductive-aged women	NCT 04177693	39 women w/wo uterine fibroids	Capsules of GTE for a daily dose of 720 mg EGCG, w/wo an ovarian stimulation medication	No subject had evidence of drug-induced liver injury or serum folate levels outside the normal range
Acosta et al. (2023) [71]	U.S.A.	To investigate the influence of COMT and UGT1A4 genotypes on changes in liver injury biomarkers	NCT 00917735	1075 healthy postmenopausal women	Capsules of GTE (EGCG 843 ± 44 mg/day) or placebo for 12 months	COMT and UGT1A4 genotypes affected serum ALT levels and AST:ALT ratios at different time intervals
Bathgate et al. (2023) [72]	U.S.A.	To test whether subjects taking EGCG supplements had reduced plasma F2-isoprostane concentrations	NCT 00917735 (secondary analysis)	A subset (N = 252) of 1075 healthy postmenopausal women	Capsules of GTE (EGCG 843 ± 44 mg/day) or placebo for 12 months	GTE supplement did not result in a significant decrease in plasma F2-isoprostanes levels. COMT genotype did not modify the effect of GTE on F2-isoprostanes
Wan et al. (2022) [73]	China	To investigate the protective effect of TP against acrylamide exposure by measuring urine and blood mercapturic acid and hemoglobin adducts of acrylamide	NCT 03118167	78 young volunteers exposed to acrylamide through potato chip consumption	Capsules containing 200 mg, 100 mg, 50 mg, or 0 mg of TP	TP supplementation attenuated the toxicity of acrylamide exposure by promoting the mercapturic acid detoxification pathway

Abbreviations: GTE, green tea extract; COMT, catechol-O-methyltransferase; UGT1A4, uridine 5′-diphospho-glucuronosyltransferase 1A4; AST, aspartate transaminase; ALT alanine transaminase; TP, tea polyphenols (total catechins ≥ 80% and EGCG ≥ 50%).

**Table 3:** Drug pharmacokinetics affected by catechins: evidence from interventional clinical studies.

Author (Year)	Country	Purpose	Trial Registry Number	Sample	Intervention	Major Findings
Misaka et al. (2022) [76]	Japan	To evaluate whether the pharmacokinetics of fexofenadine and pseudoephedrine are affected when administered orally in a GTE aqueous solution	UMIN 000032828	10 healthy volunteers	Fexofenadine and pseudoephedrine dissolved in water or in a solution containing 325 mg GTE (92.5% of EGCG)	Plasma concentrations and urinary excretions of fexofenadine were markedly decreased when co-administered with GTE
Veerman et al. (2022) [77]	The Netherlands	To study the interaction nintedanib/GTE in patients with fibrotic ILD	NL 8913	26 patients treated with nintedanib both in period A and B	500 mg GTE (60.7% EGCG) administered with 250 mL of water only in period B	Exposure to nintedanib decreased by 21% when administered 60 min after GTE for 7 days

Abbreviations: GTE, green tea extract; ILD, interstitial lung disease.

**Table 4:** Effect of catechins on cognitive functions: evidence from interventional clinical studies.

Author (Year)	Country	Purpose	Trial Reg. Identifier	Sample	Intervention	Major Findings
Uchida et al. (2024) [78]	Japan	To test the effect of matcha green tea on cognitive function and sleep quality	UMIN 000035658	99 older adults with cognitive decline or mild cognitive impairment	Daily supplementation with 2 g matcha (containing 105.3 mg of EGCG/170.8 mg catechins) or placebo for 12 months	Regular consumption of matcha could improve emotional perception and sleep quality in older adults with mild cognitive decline
Cieuta-Walti et al. (2022) [79]	Spain and France	To evaluate safety and tolerability of a dietary supplement of EGCG and if EGCG improves cognitive and functional performance in DS	NCT 03624556	66 children with DS (aged 6–12 years)	FontUp (at a daily dose of 10 mg/kg EGCG) or placebo for 6 months	EGCG was safe and well tolerated in children with DS, but the efficacy results did not support its use in this population

Abbreviations: DS, Down syndrome; FontUp, a food supplement from Grand Fontaine Laboratories.

**Table 5:** Anti-inflammatory and anti-oxidative effect of catechins: evidence from interventional clinical studies.

Author (Year)	Country	Purpose	Trial Reg. Identifier	Sample	Intervention	Major Findings
Zeng et al. (2022) [80]	China	To evaluate the adjunctive effect of EGCG solution as a coolant during scaling and root planing in the management of chronic periodontitis	ChiCTR 2000029831	15 patients with moderate to severe chronic periodontitis; bilateral maxillary teeth were randomly divided into test side and control side	On the test side, the distilled water in the ultrasonic scaler was replaced with 5 mg/mL (≈10 mM) EGCG solution	EGCG solution revealed an additional benefit on the bleeding index at the 12-week review
Zhao et al. (2022) [81]	China	To determine whether EGCG solution reduces the incidence of RID in patients undergoing radiotherapy	NCT 02580279	180 patients after breast cancer surgery and receiving radiotherapy	EGCG solution (660 μmol/L) or placebo (0.9% NaCl saline) was sprayed into the radiation field	Prophylactic use of EGCG solution significantly reduced the incidence and severity of RID
Xie et al. (2023) [82]	China	To evaluate the safety and efficacy of an EGCG solution for the treatment of acute severe dermatitis in patients receiving radiotherapy	NCT 02580279	19 patients with thoracic cancer receiving radiotherapy	EGCG solution (max. concentration: 2574 µmol/L) was sprayed into the radiation field when RID level III appeared for the first time	A decreasing trend in RID was observed, and the associated symptoms were significantly reduced
Yin et al. (2024) [83]	China	To evaluate the safety and efficacy of EGCG aerosol for the control of COVID-19 pneumonia in cancer patients	NCT 05758571	54 patients diagnosed with malignant tumor, SARS-CoV-2 infection, and moderate pneumonia	Patients were treated with EGCG nebulization (10 mL) three times daily for at least seven days; adverse events were registered	EGCG may be effective in COVID-19-induced pneumonia, promoting an improvement in moderate pneumonia or preventing the development of severe pneumonia
Cohen et al. (2024) [84]	U.S.A.	To investigate molecular and cellular basis of ILD using lung biopsy and single-cell RNA-Seq	NCT 03928847	8 ILD patients (4 in test and 4 in control group)	600 mg of EGCG by mouth once daily for 2 weeks prior to biopsy in the treatment group	EGCG downregulated TGF-β1 signaling and several pro-inflammatory and stress pathways in biopsy samples

Abbreviation: RID, radiation-induced dermatitis; ILD, interstitial lung disease.

**Table 6:** Effect of catechins on obesity and metabolism: evidence from interventional clinical studies.

Author (Year)	Country	Purpose	Trial Reg. Identifier	Sample	Intervention	Major Findings
Wilasrusmee et al. (2024) [85]	Thailand	To investigate the effects of EGCG on BP and autonomic nervous system, as indicated by 5 min heart rate variability measurement in obese subjects	TCTR 20200422001	30 obese subjects	Capsules of 150 mg EGCG (n = 15) or placebo (n = 15) twice a day without dietary restrictions	8-week EGCG treatment decreased BP and increased the LF/HF power ratio, reflecting increased sympathetic activity
Gu et al. (2023) [86]	China	To investigate the mechanism of EGCG in the prevention of obesity-related precocious puberty by means of serum metabolomics and network pharmacology	NCT 03628937	34 obese girls (6 to 10 years old)	Capsules of EGCG (200 mg, 50% EGCG, n = 18) or placebo (n = 16) for 12 weeks	EGCG may contribute to the prevention through AKT1, EGFR, ESR1, STAT3, IGF1, and MAPK1 targets and multiple signaling pathways, including the estrogen pathway
Jardon et al. (2024) [87]	The Netherlands	To investigate sex-specific differences in microbiota composition and interactions with cardiometabolic parameters after polyphenol supplementation in overweight/obese individuals	NCT 02381145	18 healthy Caucasian men and 19 women with overweight or obesity (BMI > 25 kg/m^2^)	Capsules of EGCG and resveratrol (EGCG + RES, 282 + 80 mg/d) or placebo for 12 weeks	EGCG + RES supplementation did not induce changes in the gut microbiota of men and women. Microbiota composition seemed to be predictive for polyphenol-induced changes in SkMOx in men but not in women
Churm et al. (2023) [88]	UK	To explore the impact of EGCG ingestion on catecholamine metabolism during graded cycle exercise (to exhaustion) in men	NCT 03199430	8 healthy males performing exercise 3–5 times per week (30–90 min per session)	2 capsules (1450 mg) with at least 94% EGCG and <0.1% caffeine or a placebo	Acute EGCG supplementation reduced circulating catecholamines but not metanephrine, glucose, or lactate

Abbreviation: BP, blood pressure; LF/HF, low/high frequency; RES, resveratrol; SkMOx, mitochondrial respiration in permeabilized skeletal muscle fibers.

## Data Availability

No new data were created or analyzed in this study. Data sharing is not applicable to this article.

## Abbreviations

The following abbreviations are used in this manuscript:

ABCB1	ATP-binding cassette (ABC) transporter B1
ALT	Alanine transaminase
AKT1	Serine/threonine kinase 1
AST	Aspartate transaminase
BMI	Body mass index
C	Catechin
COMT	Catechol-O-methyltransferase (COMT)
DBP	Diastolic blood pressure
DILIN	Drug-Induced Liver Injury Network
DS	Down syndrome
EC	Epicatechin
ECG	Epicatechin gallate
EFSA	European Food Safety Authority
EGC	Epigallocatechin
EGCG	Epigallocatechin-3-gallate
EGFR	Epidermal growth factor receptor
ESR1	Estrogen Receptor 1
GSH	Reduced glutathione
GSSG	Oxidized glutathione
GTE	Green tea extract
HDL-C	High-density lipoprotein cholesterol
HF	High frequency
IGF1	Insulin-like growth factor
ILD	Interstitial lung disease
IL-1β	Interleukin 1beta
IL-6	Interleukin 6
JAK	Janus kinase
LDL-C	Low-density lipoprotein cholesterol
LF	Low frequency
MAP	Mean arterial pressure
MAPK1	Mitogen-activated protein kinase 1
NCI CTCAE	National Cancer Institute Common Terminology Criteria for Adverse Events
NF-kB	Nuclear factor kappa-light-chain-enhancer of activated B cells
NLC	Nanostructured lipid carrier
OATP	Organic anion-transporting polypeptide
PI3K	Phosphoinositide 3-kinase
PLPro	Papain-like protease
RES	Resveratrol
RID	Radiation-induced dermatitis
ROS	Reactive oxygen species
SBP	Systolic blood pressure
SkMOx	Mitochondrial respiration in permeabilized skeletal muscle fibers
SLN	Solid lipid nanoparticle
STAT3	Signal transducer and activator of transcription 3
TGF-ß1	Transforming growth factor beta 1
TP	Tea Polyphenols
UGT1A4	Uridine 5′-diphospho-glucuronosyltransferase 1A4
67LR	67-kDa laminin receptor
